# Supplementary material for: Causal Effects of Modifiable Behaviors on Prostate Cancer in Europeans and East Asians: A Comprehensive Mendelian Randomization Study
Source: Biology (Basel). 2023 Apr 29;12(5):673. doi: 10.3390/biology12050673 (PMC10215492; doi:10.3390/biology12050673)
Supplement: Supplementary file 1 [file biology-12-00673-s001.zip › Supplementary Materials.pdf]

Causal effects of modifiable behaviors on prostate cancer in Europeans and East Asians: a comprehensive Mendelian randomization study

Supplementary materials:

Text S1. Detailed analytic plan 1

Table S1. Characteristics of GWAS summary data for behaviors and PCa in European ancestry 2

Table S2. Characteristics of GWAS summary data for behaviors and PCa in East Asian ancestry 4

Table S3. Univariable MR result based on IVW method in European discovery.................................. 6

Table S4. Detection of outlying SNPs in European discovery by MR-PRESSO and radial regression .. 8

Table S5. Univariable MR result based on IVW method in European replication 10

Table S6. Univariable MR result based on IVW method in East Asian discovery 12

Table S7. Univariable MR result based on IVW method in East Asian replication 13

Figure S1. Radial plots to detect outlying SNPs of the 17 traits in the European discovery sample..... 14

Figure S2. Scatter plots of the 17 behavioral factors in relation to PCa in the European discovery sample............ 15

Figure S3. Forest plots of each single SNP of the 17 behavioral factors in relation to PCa in the European discovery sample 16

Figure S4. Funnel plots of each single SNP of the 17 behavioral factors in relation to PCa in the European discovery sample 17

Figure S5. Leave-one-out analyses of the 17 behavioral factors in relation to PCa in the European discovery sample 18

Figure S6. Causal effects of modifiable behavioral factors on prostate cancer by MVMR-LASSO in Europeans and East Asians............ 19

Figure S7. Causal effects of modifiable behaviors on prostate cancer by UVMR and MVMR in European replication sample 20

Figure S8. Scatter plots of the 13 behavioral factors in relation to PCa in the East Asian discovery sample............ 21

Figure S9. Forest plots of each single SNP of the 13 behavioral factors in relation to PCa in the East Asian discovery sample 22

Figure S10. Funnel plots of each single SNP of the 13 behavioral factors in relation to PCa in the East Asian discovery sample 23

Figure S11. Leave-one-out analyses of the 12 behavioral factors in relation to PCa in the East Asian discovery sample 24

Figure S12. Causal effects of modifiable behaviors on prostate cancer by UVMR and MVMR in East Asian replication sample 25

Text S1: Detailed analytic plan

- Framework: two-sample univariable and multivariable Mendelian randomization analysis
- Total proportion of phenotypic variation explained by SNPs: R^2

  $R^2=\Sigma[2\times(1\text{-MAF})\times \text{MAF}\times\beta^2/(\text{SE}^2\times \text{N})]$

  β and SE: the coefficient and standard error for effect size

  MAF: minor allele frequency for each SNP

  N: sample size
- Instrument strength: F-statistic

  $F=(N\text{-}K\text{-}1)/K\times[R^2/(1\text{-}R^2)]$

  N: sample size

  K: total number of SNPs selected for MR analysis

  R^2: total proportion of phenotypic variation explained by all the included SNPs
- Statistical power calculation: mRnd platform (https://shiny.cnsgenomics.com/mRnd/)
- Bias estimate by sample overlapped: https://sb452.shinyapps.io/overlap/
- Primary MR analytical approach: random-effects inverse-variance weighted (IVW)
- Heterogeneity detection: Cochran's Q test with I^2 statistics
- Horizontal pleiotropy examination: MR-Egger intercept
- Outlier detection: MR-pleiotropy residual sum and outlier (MR-PRESSO), the radial regression method, and leave-one-out analyses
- Sensitivity analysis: MR-Egger regression, weighted-median, simple-mode, weighted-mode for UVMR; MR-least absolute shrinkage and selection operator (LASSO) method for MVMR
- Statistical software: All analyses were performed using the TwoSampleMR (v0.5.6), MendelianRandomization (v0.6.0), MRPRESSO (v1.0), RadialMR (v1.0) and MVMR (v0.3) packages in R4.1.2 (R Foundation for Statistical Computing, Vienna, Austria).

.

Table S1. Characteristics of GWAS summary data for behaviors and PCa in European ancestry

Traits	GWAS ID	PubMed ID	Year	Sample size	No. SNPs	Units	Author	Consortium
**Prostate cancer**								
Discovery	ieu-b-85	29892016	2018	Cases: 79,148<br>Total: 140,254	20,346,368	N/A	Schumacher FR	PRACTICAL
Replication	finn-b-C3_PROSTATE	N/A	2021	Cases: 6,311<br>Total: 95,213	16,378,835	N/A	N/A	FinnGen
**Tobacco smoking**								
Ever smoking	ukb-b-20261	N/A	2018	461,066	9,851,867	N/A	Elsworth B	MRC-IEU
Smoking initiation	ieu-b-4877	30643251	2019	632,802	11,802,365	N/A	Liu M	GSCAN
Lifetime smoking index	N/A	31689377	2020	462,690	7,683,478	N/A	Wootton RE	UK Biobank
**Alcohol drinking**								
Alcohol frequency	ukb-b-5779	N/A	2018	462,346	9,851,867	SD	Elsworth B	MRC-IEU
Alcohol quantity	ieu-b-73	30643251	2019	335,394	11,887,865	SD	Liu M	GSCAN
**Fruit intake**								
Fresh fruit	ukb-b-3881	N/A	2018	446,462	9,851,867	SD	Elsworth B	MRC-IEU
Dried fruit	ukb-b-16576	N/A	2018	421,764	9,851,867	SD	Elsworth B	MRC-IEU
**Vegetables intake**								
Raw vegetable	ukb-b-1996	N/A	2018	435,435	9,851,867	SD	Elsworth B	MRC-IEU
Cooked vegetable	ukb-b-8089	N/A	2018	448,651	9,851,867	SD	Elsworth B	MRC-IEU
**Physical activity**								
Duration of vigorous activity	ukb-b-13932	N/A	2018	251,501	9,851,867	SD	Elsworth B	MRC-IEU
Duration of moderate activity	ukb-b-2346	N/A	2018	343,827	9,851,867	SD	Elsworth B	MRC-IEU

Duration of light activity	ukb-b-8865	N/A	2018	64,949	9,851,867	SD	Elsworth B	MRC-IEU
**Sexual behavior**								
Age of initiation	ebi-a-GCST90000047	34211149	2021	397,338	16,359,424	N/A	Mills MC	Human Reproductive Behaviour Consortium
No. of sexual partner	ukb-b-4256	N/A	2018	378,882	9,851,867	SD	Elsworth B	MRC-IEU
**Sleep behavior**								
Insomnia	ukb-b-3957	N/A	2018	462,341	9,851,867	SD	Elsworth B	MRC-IEU
Nap during day	ebi-a-GCST011494	33568662	2021	452,633	13,024,091	N/A	Dashti HS	UK Biobank and GIANT consortium
Daytime dozing	ukb-b-5776	N/A	2018	460,913	9,851,867	N/A	Elsworth B	MRC-IEU

GIANT, the Genetic Investigation of ANthropometric Traits; GSCAN, GWAS and Sequencing Consortium of Alcohol and Nicotine use; MRC-IEU, the Medical Research Council - Integrative Epidemiology Unit; PRACTICAL, Prostate Cancer Association Group to Investigate Cancer-Associated Alterations in the Genome; N/A, not applicable

Table S2. Characteristics of GWAS summary data for behaviors and PCa in East Asian ancestry

Traits	GWAS ID	Year	Sample size	SNPs (n)	Author	Consortium
**Prostate cancer**						
Discovery	N/A	2022	Cases: 3,343<br>Total: 6,658	10,661,929	ChinaPCa	ChinaPCa
Replication	bbj-a-148	2019	Cases: 5,408<br>Total: 109,347	8,878,753	Ishigaki K	Biobank Japan
**Tobacco smoking**						
Ever smoking	ukb-e-20160_EAS	2020	2,649	82,146,731	Pan-UKB team	Pan-UKB
**Alcohol drinking**						
Alcohol frequency	ukb-e-1558_EAS	2020	2,658	8,256,422	Pan-UKB team	Pan-UKB
**Fruit intake**						
Fresh fruit	ukb-e-1309_EAS	2020	2,513	8,255,183	Pan-UKB team	Pan-UKB
Dried fruit	ukb-e-1319_EAS	2020	2,219	8,275,593	Pan-UKB team	Pan-UKB
**Vegetables intake**						
Raw vegetable	ukb-e-1299_EAS	2020	2,365	8,268,823	Pan-UKB team	Pan-UKB
Cooked vegetable	ukb-e-1289_EAS	2020	2,499	8,257,733	Pan-UKB team	Pan-UKB
**Physical activity**						
Duration of vigorous activity	ukb-e-914_EAS	2020	1,373	8,283,408	Pan-UKB team	Pan-UKB
Duration of moderate activity	ukb-e-894_EAS	2020	1,853	8,264,440	Pan-UKB team	Pan-UKB
**Sexual behavior**						
Age of initiation	ukb-e-2139_EAS	2020	1,711	8,345,332	Pan-UKB team	Pan-UKB
No. of sexual partner	ukb-e-2149_EAS	2020	1,559	8,326,030	Pan-UKB team	Pan-UKB

**Sleep behavior**						
Insomnia	ukb-e-1200_EAS	2020	2,654	8,256,478	Pan-UKB team	Pan-UKB
Nap during day	ukb-e-1190_EAS	2020	2,606	8,258,355	Pan-UKB team	Pan-UKB
Daytime dozing	ukb-e-1220_EAS	2020	2,582	8,260,991	Pan-UKB team	Pan-UKB

Table S3. Univariable MR result based on IVW method in European discovery

Factors	Effect size		Heterogeneity			Pleiotropy	
	OR	P	Q-stat	P	I^2 (%)	MR-Egger intercept	P
**Tobacco smoking**							
Ever smoking	1.05 (0.75, 1.48)	0.770	101.85	**0.018**	27.35	0.00	0.666
Smoking initiation	0.92 (0.82, 1.02)	0.118	173.33	**<0.001**	51.54	0.01	0.373
Lifetime smoking index	**0.75 (0.58, 0.97)**	**0.026**	245.41	**<0.001**	57.62	0.01	0.135
**Alcohol drinking**							
Alcohol frequency	**0.89 (0.80, 0.99)**	**0.038**	149.60	**<0.001**	45.86	0.00	0.549
Alcohol quantity	0.94 (0.60, 1.48)	0.787	161.06	**<0.001**	80.13	-0.01	0.460
**Fruit intake**							
Fresh fruit	1.25 (0.83, 1.88)	0.283	92.16	**<0.001**	51.17	0.01	0.327
Dried fruit	1.15 (0.70, 1.89)	0.573	127.57	**<0.001**	73.35	0.02	0.191
**Vegetable intake**							
Raw vegetable	2.15 (0.87, 5.31)	0.096	54.90	**<0.001**	72.68	-0.01	0.833
Cooked vegetable	1.25 (0.61, 2.55)	0.536	38.52	**<0.001**	63.65	0.05	0.265
**Physical activity**							
Duration of vigorous activity	1.04 (0.54, 1.99)	0.903	0.98	0.612	0	0.04	0.508
Duration of moderate activity	1.24 (0.60, 2.57)	0.562	1.10	0.576	0	0.02	0.595
Duration of light activity	1.00 (1.00, 1.01)	0.468	1.78	0.411	0	0.03	0.650
**Sexual behavior**							
Age of initiation	**1.18 (1.01, 1.38)**	**0.035**	325.73	**<0.001**	58.55	<0.001	0.954
No. of sexual partners	0.90 (0.64, 1.26)	0.532	184.51	**<0.001**	70.19	-0.01	0.551
**Sleep behavior**							

Insomnia	1.17 (0.80, 1.71)	0.429	60.98	**0.001**	47.53	0.01	0.244
Nap during day	0.97 (0.77, 1.24)	0.825	125.90	**0.011**	26.93	0.00	0.861
Daytime dozing	0.90 (0.44, 1.87)	0.784	61.04	**<0.001**	59.04	-0.01	0.855

Table S4. Detection of outlying SNPs in European discovery by MR-PRESSO and radial regression

Factors	MR-PRESSO				Radial regression			
	**Outlier**	**OR**	**95%CI**	***P* value**	**Outlier**	**OR**	**95%CI**	***P* value**
**Tobacco smoking**								
Ever smoking	rs56166763<br>rs2516436	1.11	0.81-1.53	0.524	rs56166763	1.12	0.83-1.50	0.452
Smoking initiation	rs13145728	0.94	0.85-1.04	0.237	rs13145728	0.93	0.84-1.03	0.175
Lifetime smoking index	rs6778080<br>rs7077678	0.77	0.61-0.97	**0.029**	rs6778080<br>rs7077678	0.75	0.59-0.95	**0.021**
**Alcohol drinking**								
Alcohol frequency	rs8614	0.91	0.82-1.01	0.068	N/A			
Alcohol quantity	rs28929474<br>rs4752999<br>rs6739804	0.95	0.72-1.25	0.711	rs28929474<br>rs4752999<br>rs6739804	0.95	0.66-1.35	0.770
**Fruit intake**								
Fresh fruit	rs12044599	1.09	0.77-1.53	0.632	rs12044599	1.13	0.79-1.62	0.517
Dried fruit	rs11811826<br>rs4269101<br>rs429358<br>rs62084586	1.45	1.04-2.03	**0.038**	rs11811826<br>rs4269101<br>rs429358<br>rs62084586	1.48	1.06-2.06	**0.030**
**Vegetable intake**								
Raw vegetable	rs3095337<br>rs3129962	1.82	1.08-3.08	**0.041**	rs3095337<br>rs3129962<br>rs8130508	1.48	0.87-2.52	0.171

	rs8130508							
	rs9427220							
Cooked vegetable	rs2844672	0.91	0.63-1.31	0.610	rs2844672	0.91	0.62-1.31	0.611
**Physical activity**								
Duration of vigorous activity	N/A				N/A			
Duration of moderate activity	N/A				N/A			
Duration of light activity	N/A				N/A			
**Sexual behavior**								
Age of initiation	rs28929474				rs28929474			
	rs62177795	1.15	11.03-1.28	**0.015**	rs62177795	1.21	1.05-1.38	**0.008**
	rs7618715				rs7618715			
	rs809955				rs807478			
No. of sexual partner	rs2726461	0.92	0.70-1.21	0.551	rs2726461	0.94	0.71-1.26	0.696
	rs6908726				rs6908726			
**Sleep behavior**								
Insomnia	N/A				N/A			
Nap during day	N/A				N/A			
Daytime dozing	rs553314	0.67	0.36-1.26	0.231	rs553314	0.67	0.35-1.30	0.245
	rs6923811				rs6923811			

Table S5. Univariable MR result based on IVW method in European replication

Factors	Effect size		Heterogeneity			Pleiotropy	
	OR	P	Q-stat	P	I^2 (%)	MR-Egger intercept	P
**Tobacco smoking**							
Ever smoking	1.31 (0.61-2.79)	0.485	80.17	0.292	7.70	-0.01	0.802
Smoking initiation	0.90 (0.73, 1.12)	0.335	97.04	0.082	18.59	-0.01	0.706
Lifetime smoking index	1.08 (0.71, 1.63)	0.720	107.91	0.618	0.00	0.00	0.643
**Alcohol drinking**							
Alcohol frequency	1.05 (0.82, 1.33)	0.704	128.08	**0.006**	28.95	0.00	0.725
Alcohol quantity	0.88 (0.39, 2.01)	0.765	69.33	**<0.001**	56.73	-0.03	0.172
**Fruit intake**							
Fresh fruit	2.32 (0.97, 5.52)	0.058	78.96	**0.006**	36.68	-0.02	0.185
Dried fruit	1.38 (0.67, 2.85)	0.378	51.63	**0.044**	30.27	0.00	0.975
**Vegetable intake**							
Raw vegetable	**5.44 (1.20, 24.70)**	**0.028**	25.35	0.064	36.89	0.02	0.547
Cooked vegetable	3.25 (0.64, 16.62)	0.157	36.62	**0.001**	59.04	-0.02	0.841
**Physical activity**							
Duration of vigorous activity	1.29 (0.25, 6.56)	0.762	0.03	0.984	0.00	-0.02	0.888
Duration of moderate activity	**0.11 (0.01, 0.88)**	**0.037**	1.45	0.484	0.00	0.02	0.849
Duration of light activity	1.00 (0.99, 1.00)	0.295	1.06	0.590	0.00	0.10	0.492
**Sexual behavior**							
Age of initiation	0.83 (0.63, 1.09)	0.187	169.82	**0.034**	18.74	0.00	0.886
No. of sexual partner	1.11 (0.66, 1.89)	0.691	69.01	0.097	20.30	-0.02	0.223
**Sleep behavior**							

Insomnia	0.97 (0.50, 1.86)	0.922	36.68	0.437	1.86	0.00	0.989
Nap during day	1.44 (0.81, 2.57)	0.215	117.01	**0.035**	22.23	0.00	0.809
Daytime dozing	2.24 (0.55, 9.09)	0.257	38.97	0.064	30.72	0.04	0.217

Table S6. Univariable MR result based on IVW method in East Asian discovery

Factors	Effect size		Heterogeneity			Pleiotropy	
	OR	P	Q-stat	P	I^2 (%)	MR-Egger intercept	P
**Tobacco smoking**							
Ever smoking	1.05 (0.94, 1.17)	0.395	9.41	0.400	4.33	-0.04	0.742
**Alcohol drinking**							
Alcohol frequency	**1.11 (1.00, 1.23)**	**0.050**	1.80	0.773	0.00	-0.01	0.887
**Fruit intake**							
Fresh fruit	1.01 (0.73, 1.39)	0.971	5.96	0.311	16.05	-0.07	0.316
Dried fruit	1.06 (0.84, 1.34)	0.635	16.52	0.086	39.46	-0.04	0.672
**Vegetable intake**							
Raw vegetable	0.89 (0.68, 1.17)	0.416	0.64	0.727	0.00	-0.04	0.730
Cooked vegetable	1.04 (0.88, 1.23)	0.627	31.60	**0.017**	46.20	-0.01	0.800
**Physical activity**							
Duration of vigorous activity	0.93 (0.82, 1.06)	0.259	3.68	0.298	18.6	0.09	0.219
Duration of moderate activity	1.04 (0.88, 1.22)	0.801	0.12	0.944	0.00	0.02	0.835
**Sexual behavior**							
Age of initiation	0.93 (0.73, 1.19)	0.586	3.37	0.339	10.86	1.01	0.240
No. of sexual partners	0.84 (0.59, 1.21)	0.353	0.85	0.932	0.00	0.08	0.609
**Sleep behavior**							
Insomnia	0.86 (0.69, 1.07)	0.164	28.18	**0.043**	39.68	0.01	0.777
Nap during day	1.12 (0.98, 1.28)	0.104	8.85	0.784	0.00	-0.01	0.947
Daytime dozing	0.90 (0.66, 1.24)	0.535	12.13	0.595	0.00	0.03	0.520

Table S7. Univariable MR result based on IVW method in East Asian replication

Factors	Effect size		Heterogeneity			Pleiotropy	
	OR	P	Q-stat	P	I^2 (%)	MR-Egger intercept	P
**Tobacco smoking**							
Ever smoking	0.99 (0.93, 1.06)	0.795	2.67	0.849	0.00	-0.06	0.436
**Alcohol drinking**							
Alcohol frequency	1.00 (0.94, 1.06)	0.887	0.19	0.979	0.00	-0.02	0.769
**Fruit intake**							
Fresh fruit	0.98 (0.84, 1.14)	0.797	2.70	0.609	0.00	0.01	0.713
Dried fruit	0.96 (0.80, 1.16)	0.666	26.20	**<0.001**	69.46	0.02	0.817
**Vegetable intake**							
Raw vegetable	1.08 (0.94, 1.24)	0.309	<0.01	1.000	0.00	0.00	0.993
Cooked vegetable	0.97 (0.91, 1.04)	0.386	13.22	0.721	0.00	0.03	0.248
**Physical activity**							
Duration of vigorous activity	0.96 (0.89, 1.03)	0.275	2.50	0.475	0.00	-0.01	0.876
Duration of moderate activity	0.96 (0.86, 1.08)	0.498	0.93	0.628	0.00	-0.04	0.516
**Sexual behavior**							
Age of initiation	0.97 (0.84, 1.12)	0.669	4.45	0.216	32.65	-0.51	0.242
No. of sexual partner	1.05 (0.86, 1.27)	0.637	5.09	0.278	21.48	0.03	0.761
**Sleep behavior**							
Insomnia	0.97 (0.88, 1.08)	0.589	10.99	0.687	0.00	-0.03	0.270
Nap during day	0.98 (0.89, 1.08)	0.669	17.05	0.148	29.60	-0.01	0.812
Daytime dozing	0.99 (0.85, 1.16)	0.929	25.63	**0.042**	41.48	0.00	0.926

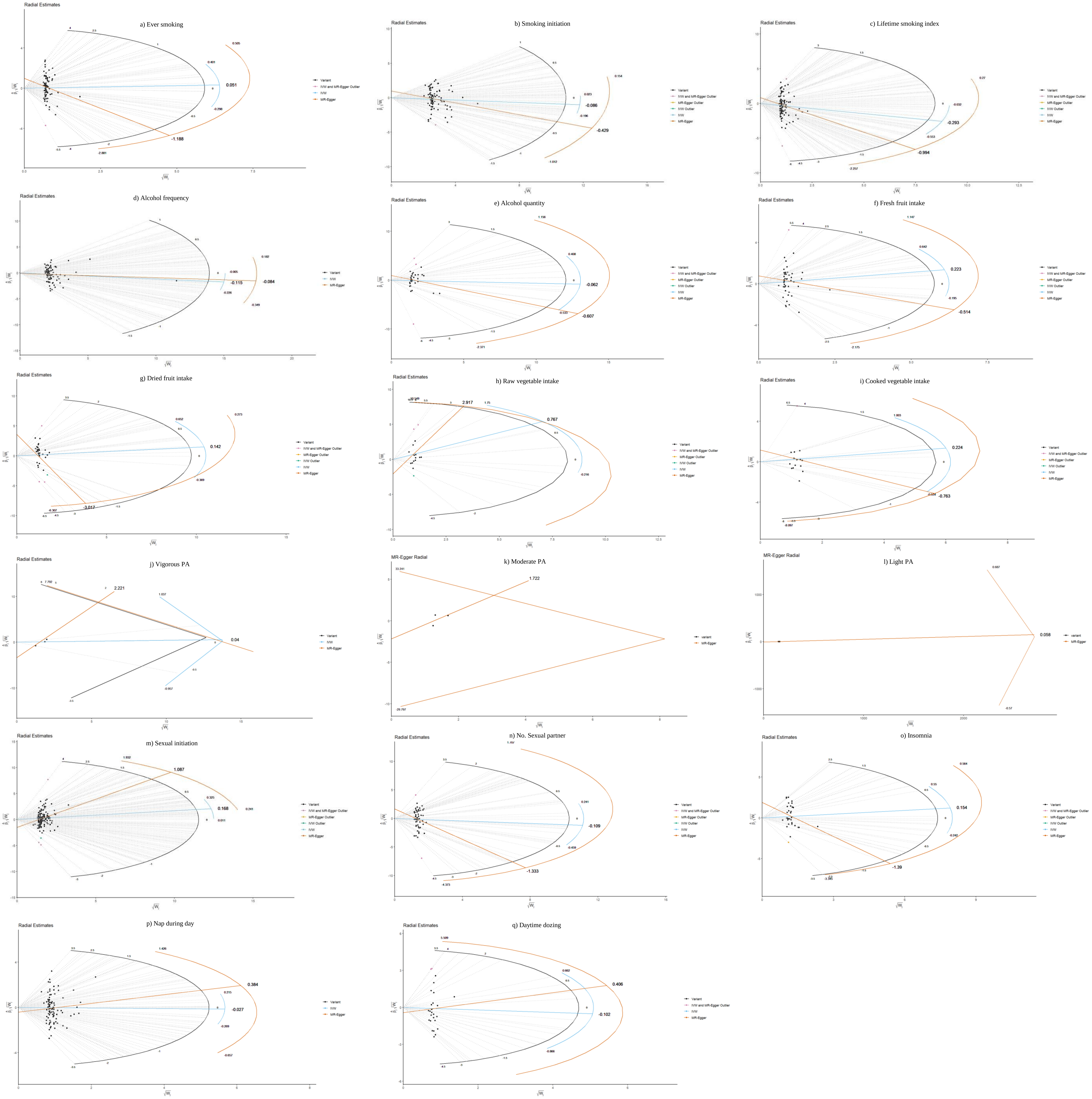

Figure S1. Radial plots to detect outlying SNPs of the 15 traits in the European discovery sample

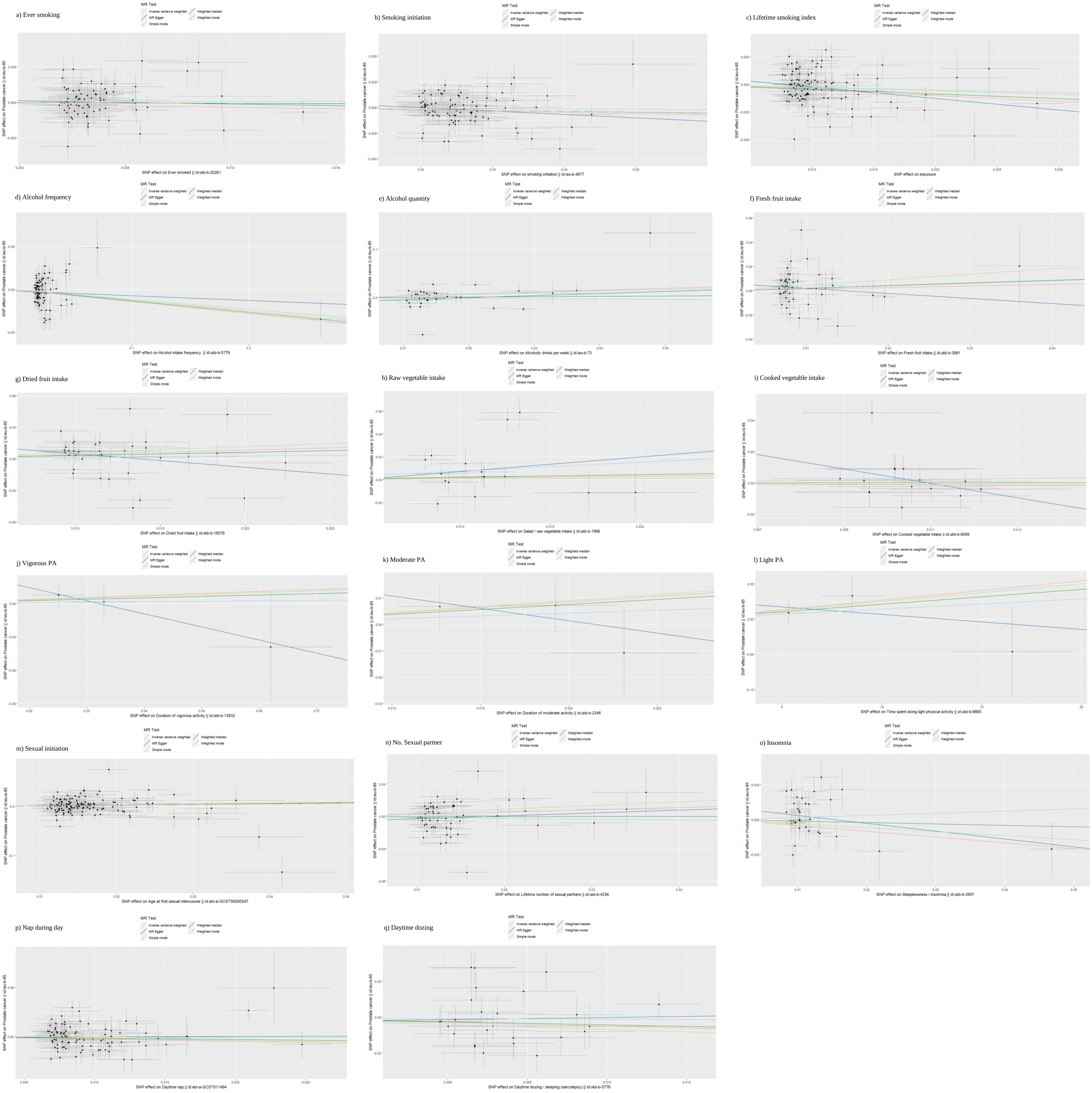

Figure S2. Scatter plots of the 15 behavioral factors in relation to PCa in the European discovery sample

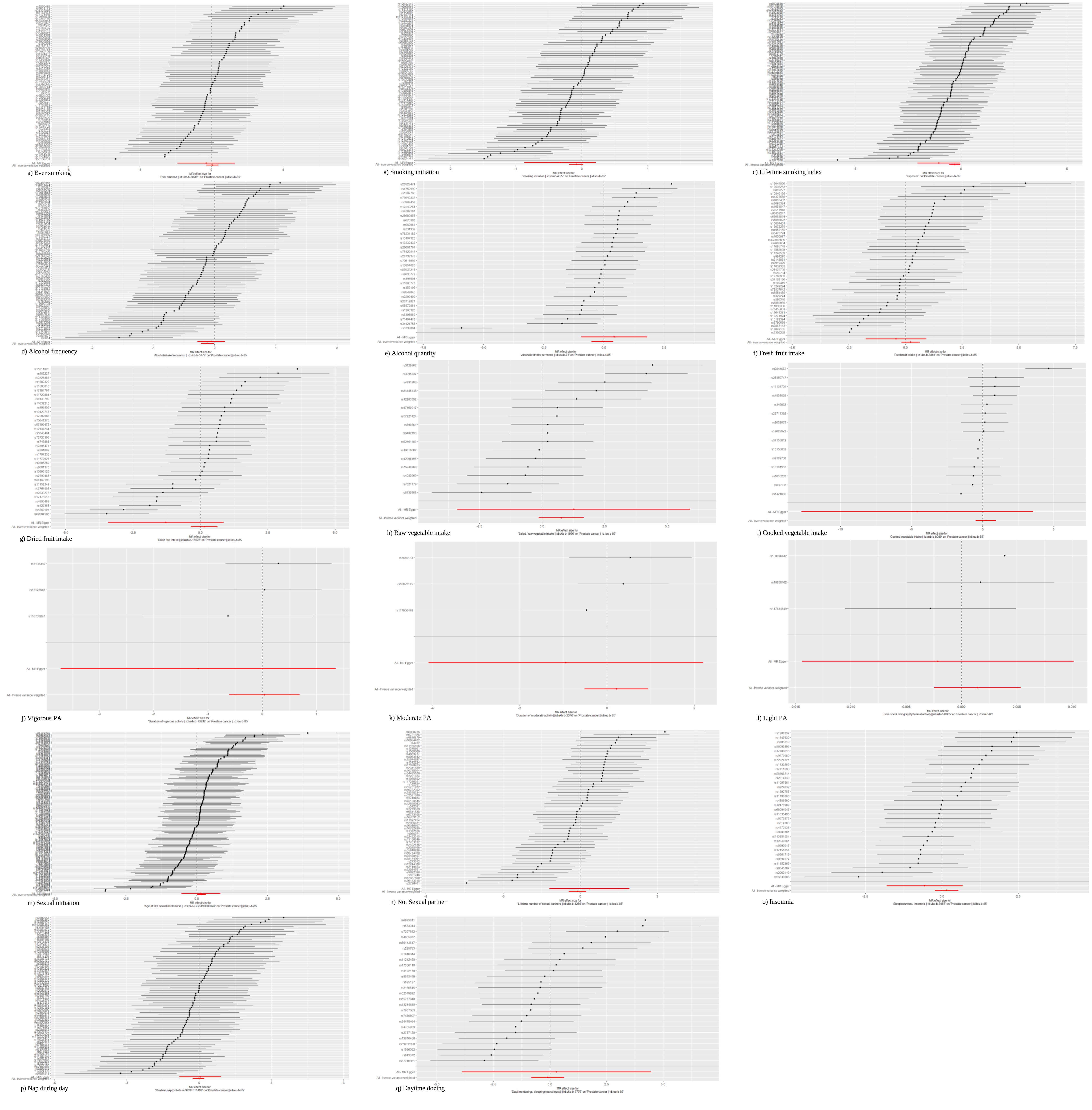

Figure S3. Forest plots of each single SNP of the 15 behavioral factors in relation to PCa in the European discovery sample

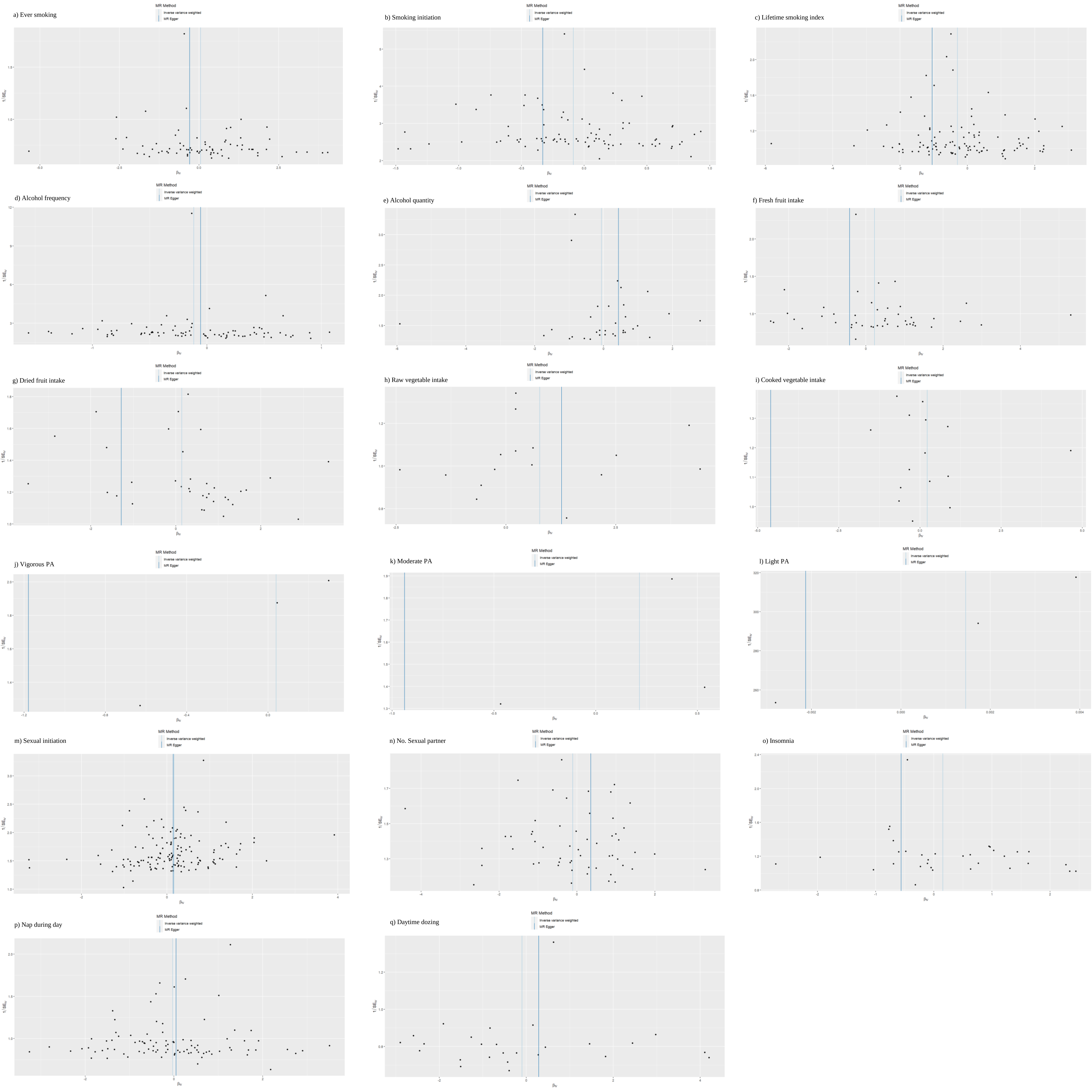

Figure S4. Funnel plots of each single SNP of the 15 behavioral factors in relation to PCa in the European discovery sample

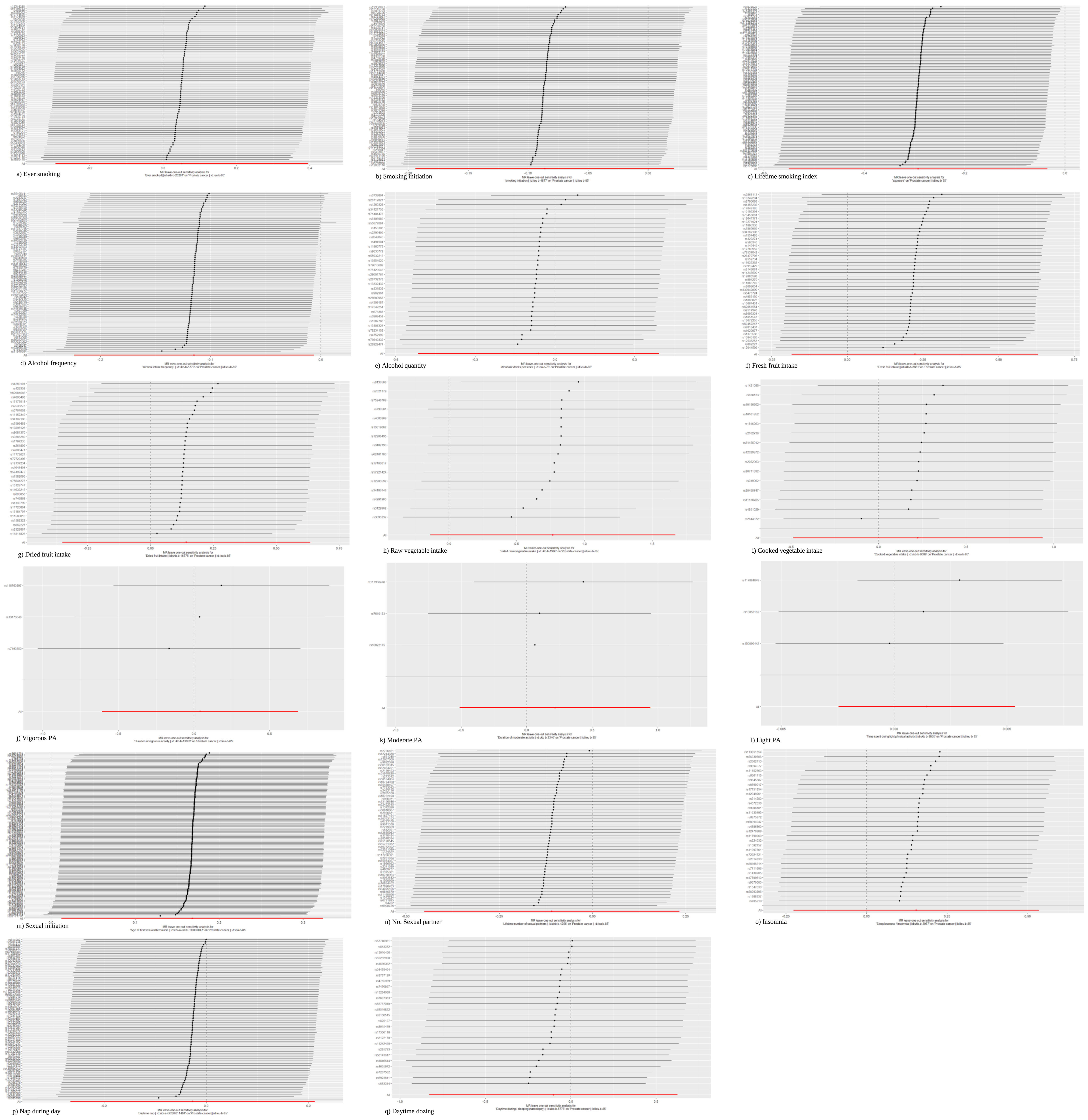

Figure S5. Leave-one-out analyses of the 15 behavioral factors in relation to PCa in the European discovery sample

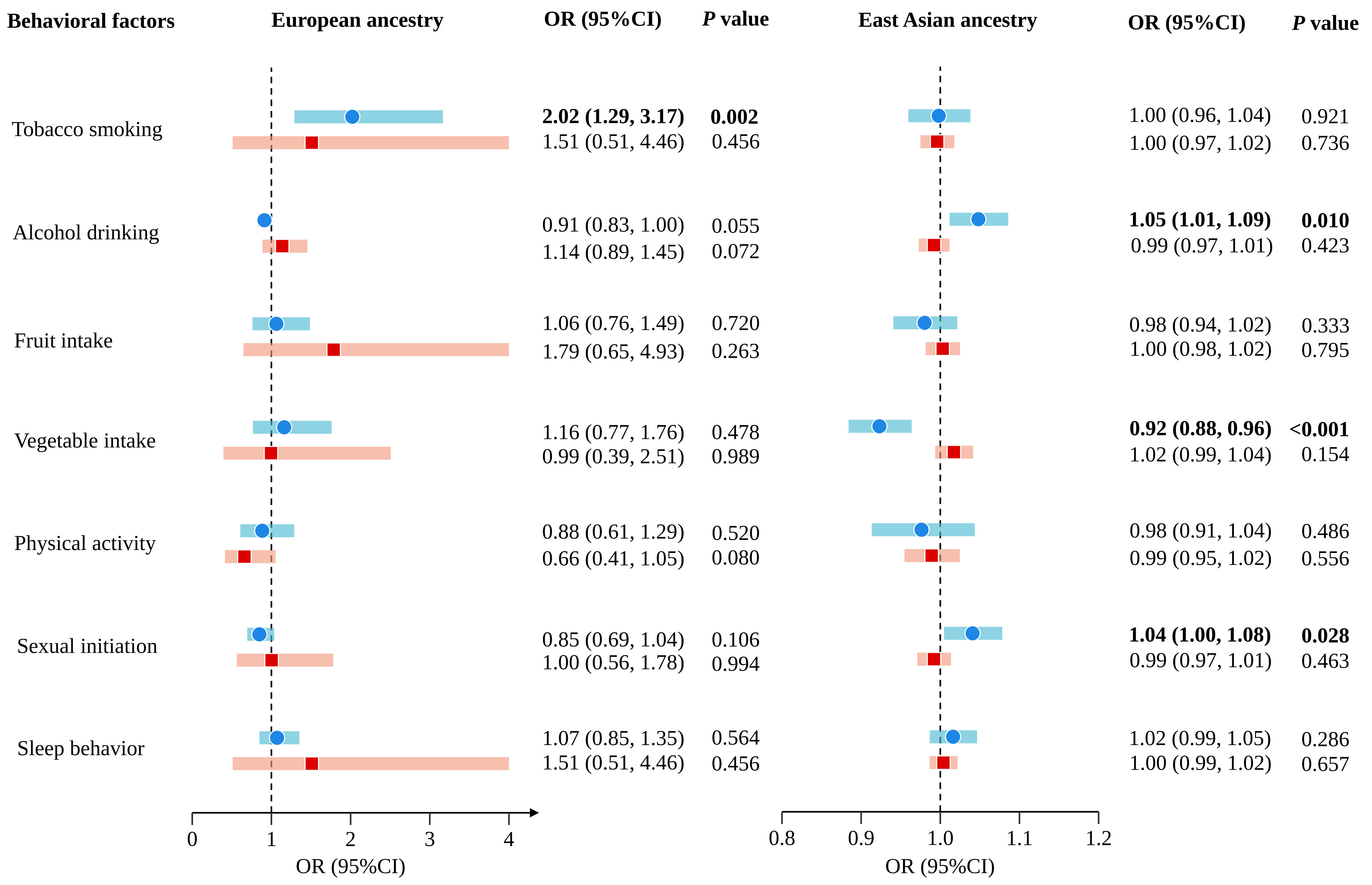

Figure S6. Causal effects of modifiable behavioral factors on prostate cancer by MVMR-LASSO in Europeans and East Asians

Discovery Replication

Behavioral factors	OR (95%CI)	*P*	OR (95%CI)	*P*
Tobacco smoking				
Ever smoking	1.31 (0.61-2.79)	0.485		
Smoking initiation	0.90 (0.73, 1.12)	0.335		
Comprehensive smoking index	1.08 (0.71, 1.63)	0.720	1.85 (0.59, 5.75)	0.291
Alcohol drinking				
Alcohol frequency	1.05 (0.82, 1.33)	0.704	1.15 (0.89, 1.48)	0.291
Alcohol quantity	0.88 (0.39, 2.01)	0.765		
Fruit intake				
Fresh fruit	2.32 (0.97, 5.52)	0.058	1.86 (0.80, 4.34)	0.154
Dried fruit	1.38 (0.67, 2.85)	0.378		
Vegetable intake				
Raw vegetable	**5.44 (1.20, 24.70)**	**0.028**	2.13 (0.75, 6.07)	0.159
Cooked vegetable	3.25 (0.64, 16.62)	0.157		
Physical activity				
Duration of vigorous activity	1.29 (0.25, 6.56)	0.762		
Duration of moderate activity	**0.11 (0.01, 0.88)**	**0.037**	1.55 (0.60, 4.02)	0.369
Duration of light activity	1.00 (0.99, 1.00)	0.295		
Sexual behavior				
Age of initiation	0.83 (0.63, 1.09)	0.187	0.67 (0.41, 1.10)	0.114
No. of sexual partner	1.11 (0.66, 1.89)	0.691		
Sleep behavior				
Insomnia	0.97 (0.50, 1.86)	0.922	0.93 (0.51, 1.68)	0.808
Nap during day	1.44 (0.81, 2.57)	0.215		
Daytime dozing	2.24 (0.55, 9.09)	0.257		

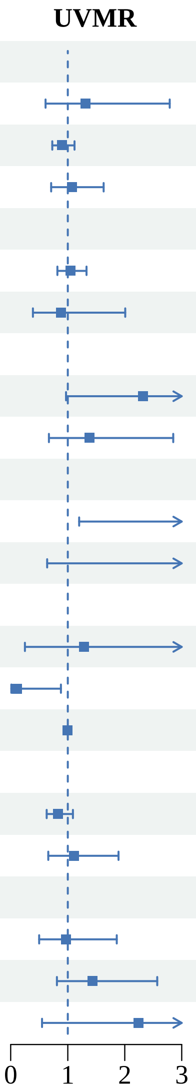

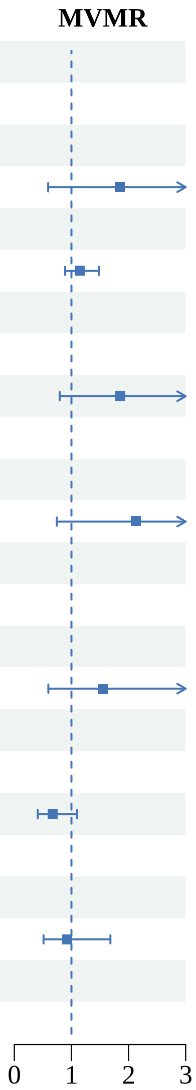

Figure S7. Causal effects of modifiable behaviors on prostate cancer by UVMR and MVMR in European replication sample

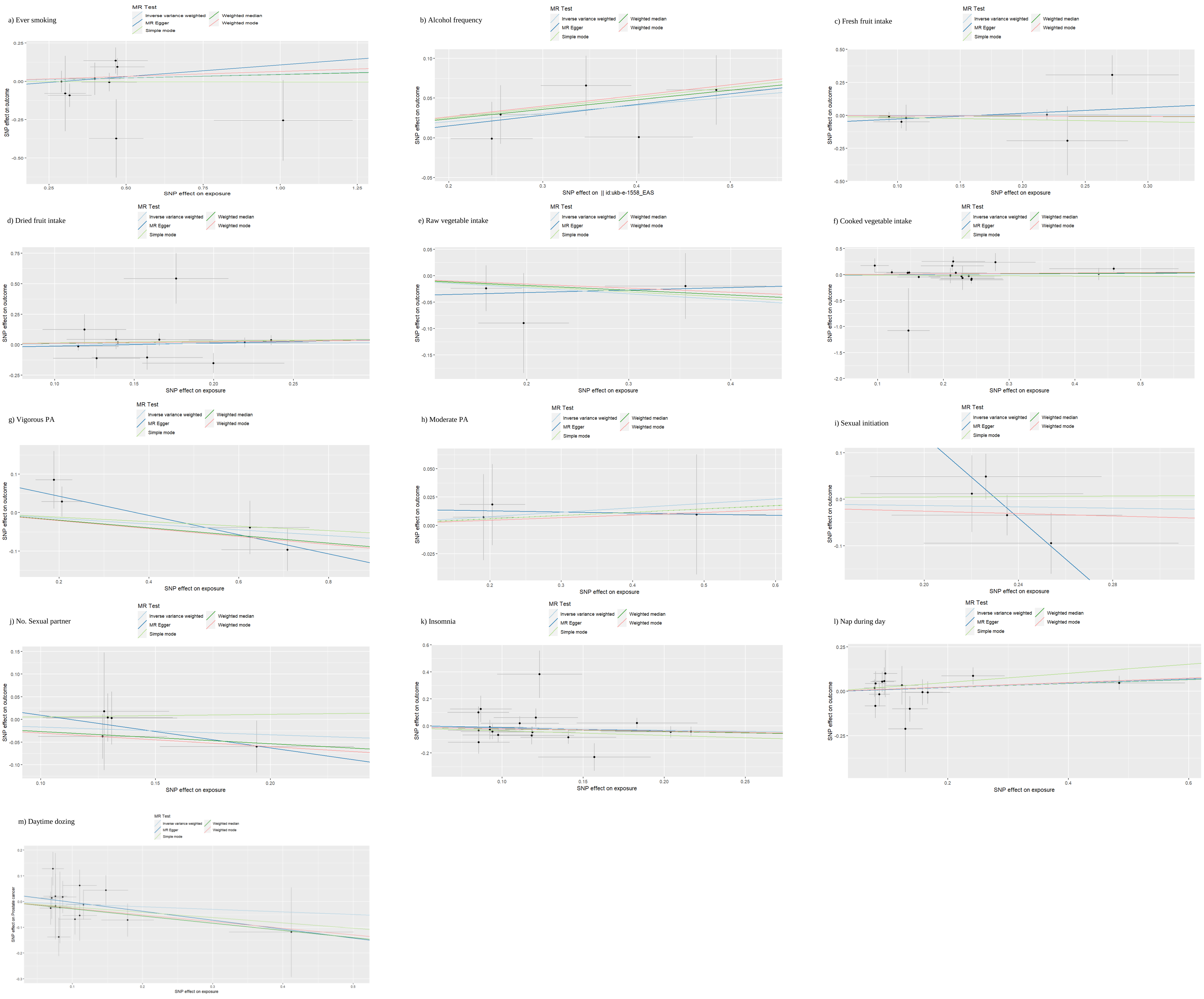

Figure S8. Scatter plots of the 12 behavioral factors in relation to PCa in the East Asian discovery sample

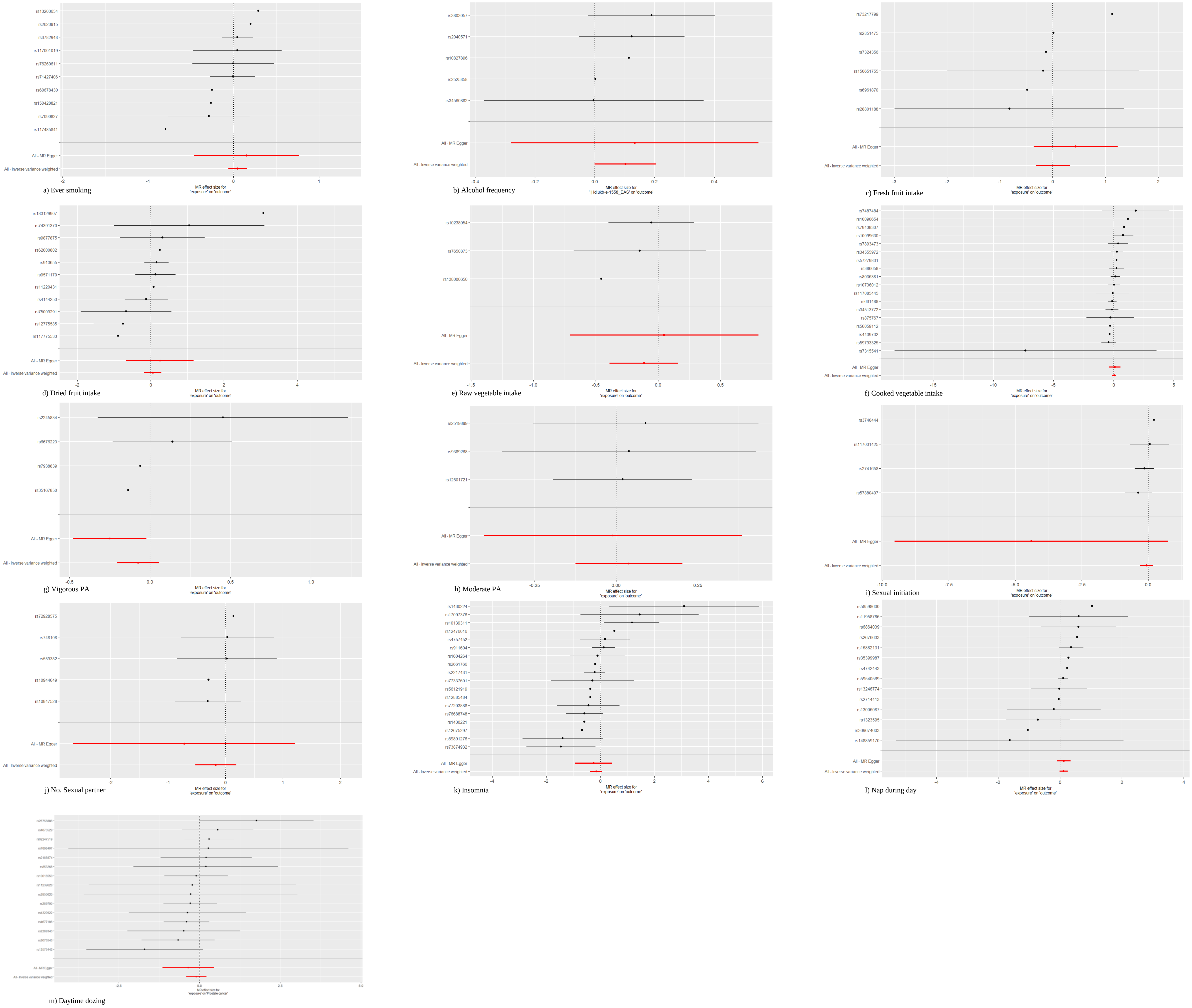

Figure S9. Forest plots of each single SNP of the 12 behavioral factors in relation to PCa in the East Asian discovery sample

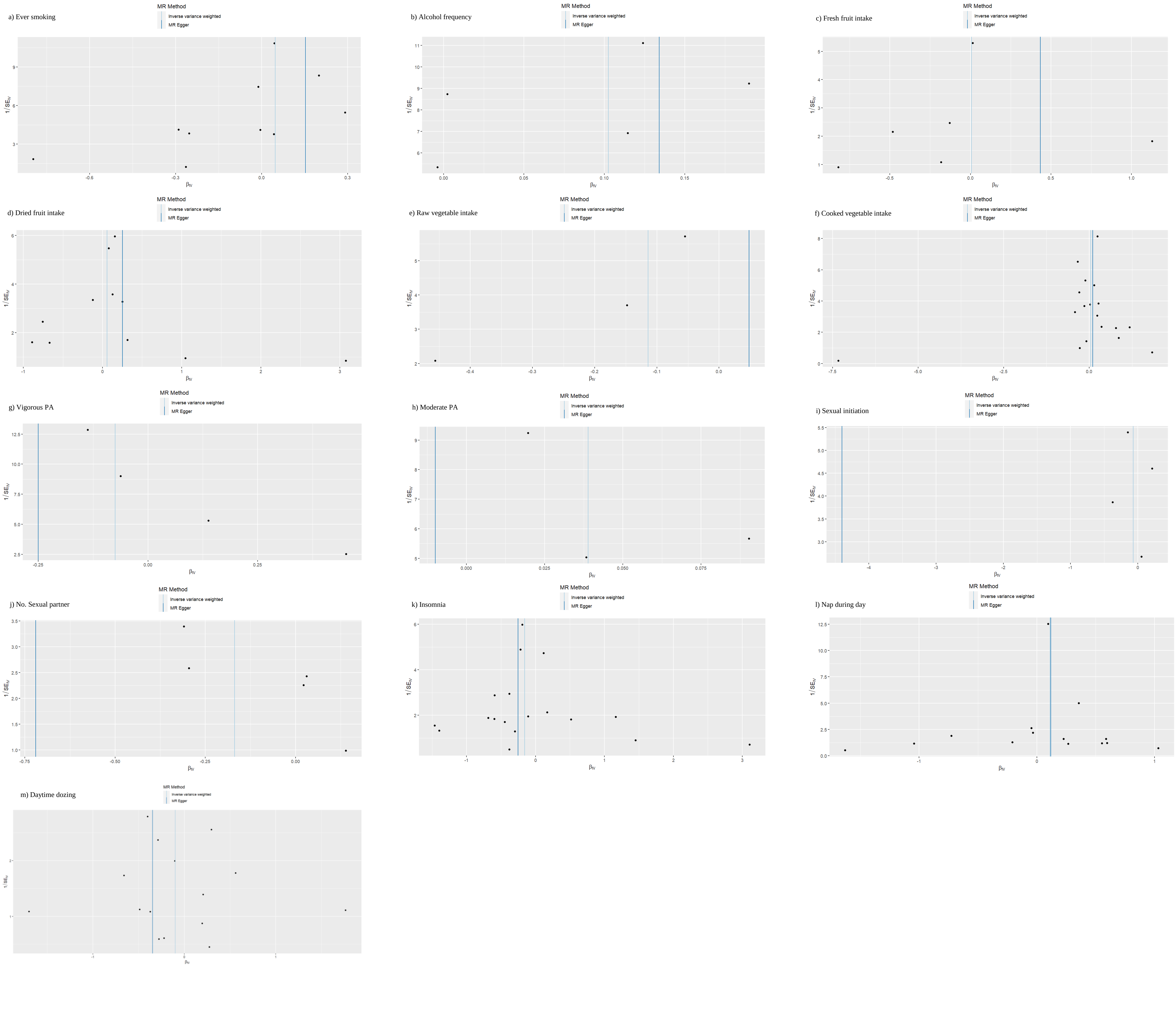

Figure S10. Funnel plots of each single SNP of the 12 behavioral factors in relation to PCa in the East Asian discovery sample

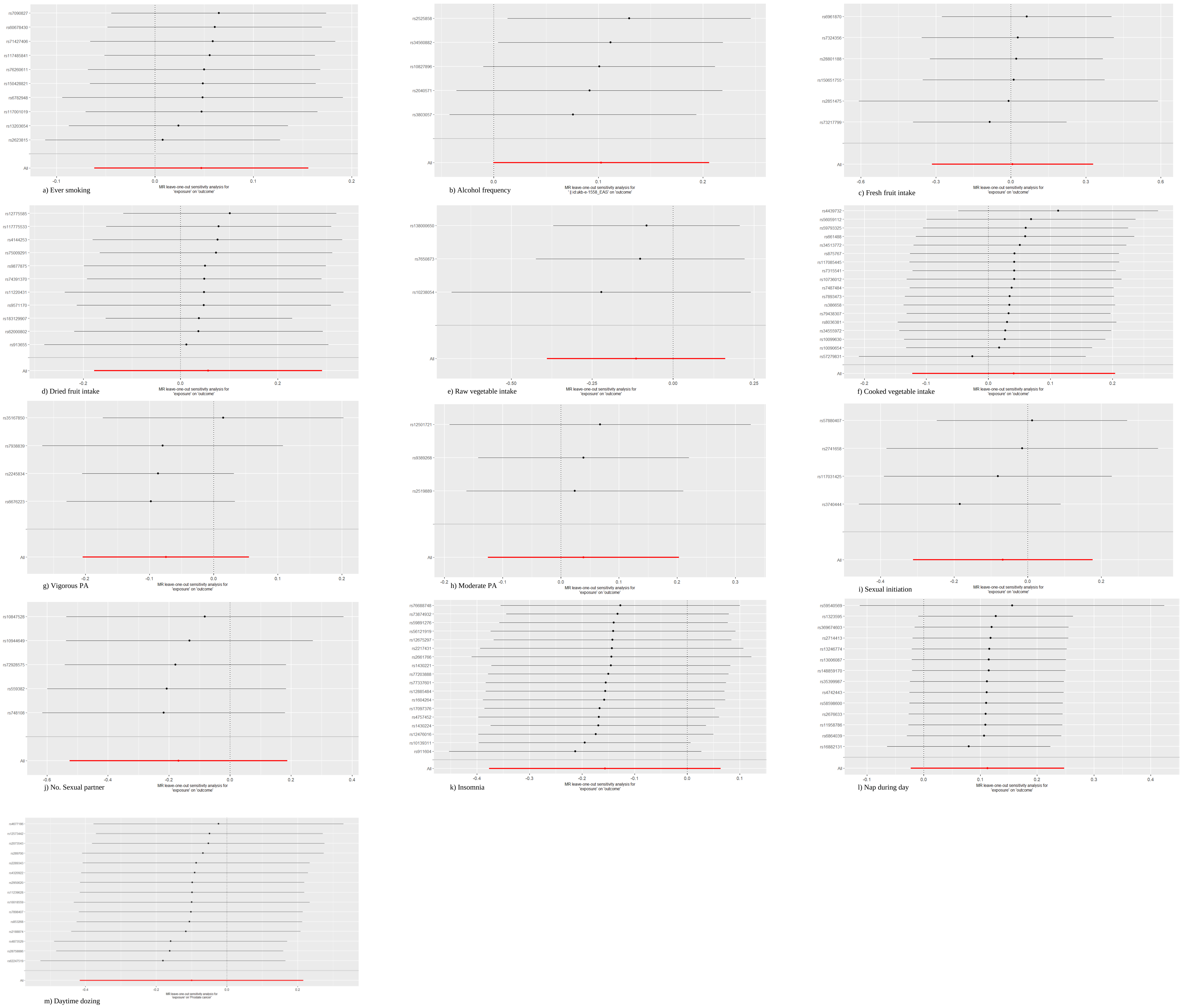

Figure S11. Leave-one-out analyses of the 12 behavioral factors in relation to PCa in the East Asian discovery sample

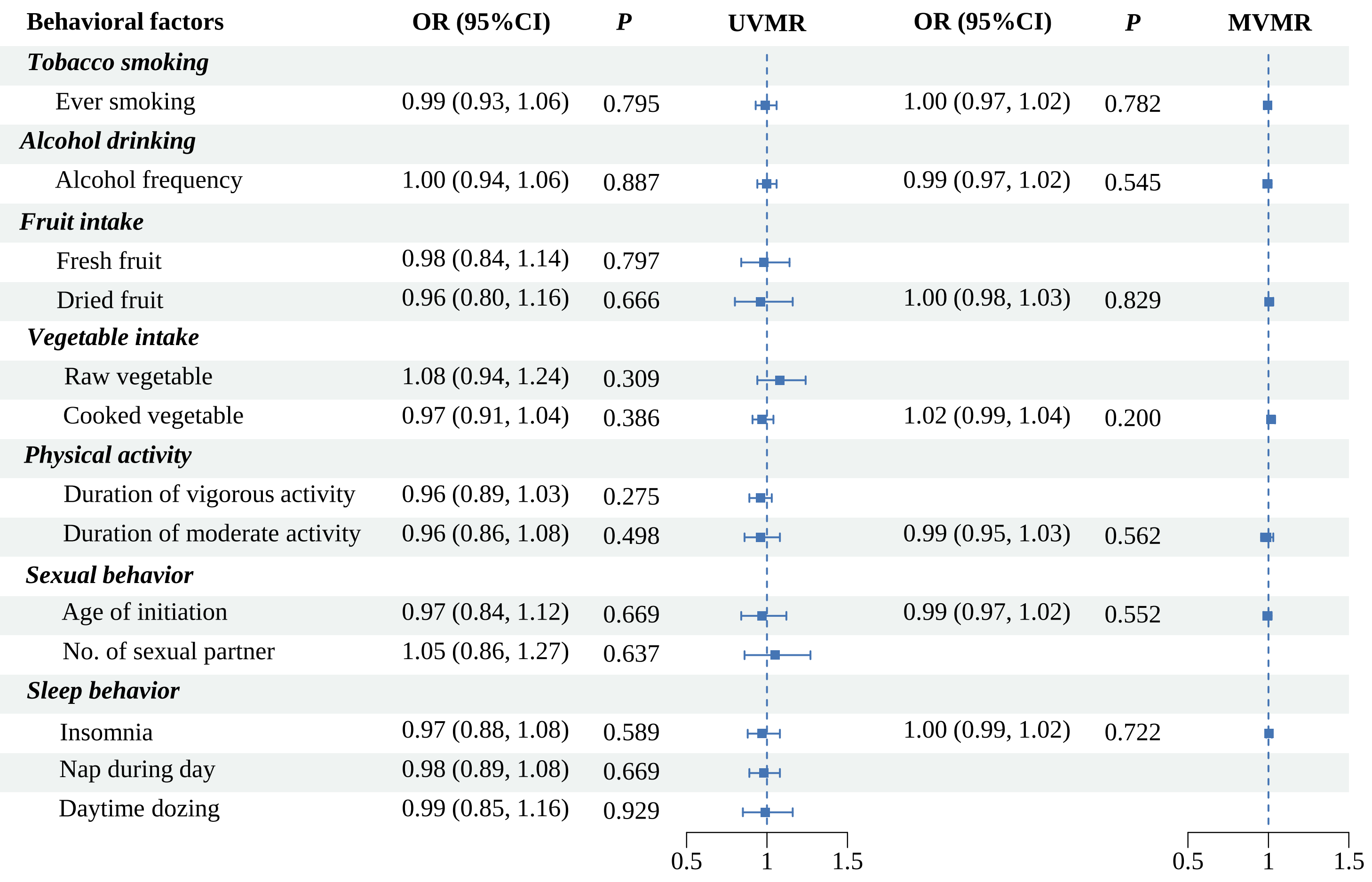

Figure S12. Causal effects of modifiable behaviors on prostate cancer by UVMR and MVMR in East Asian replication sample
